# The Effects of Indulgence in Alcohol

**Published:** 1919-02-08

**Authors:** 


### PROHIBITION IN THE U.S.A.

### The Effects of Indulgence in Alcohol. (*Communicated.*)

THAT America has "gone dry," or is likely soon so to do, is a remarkable social revolution which must have its reflex in Great Britain. Even before this portentous news came it was being urged that at least the war-time restrictions on the sale of alcoholic liquors should be continued after peace, on the grounds that public health and the general well-being of the community have been bettered thereby. The success of the prohibition propaganda in America will hearten agitation in Britain.

The use of alcohol is a subject seldom debated without prejudice and heat; an attitude as much due to the foolishness of the folk who succeeded in establishing themselves as the Teetotal party as to any other cause. The manners and methods of a large section of the reformers were offensive and brought them and their cause into contempt. Intemperate abuse provoked abusive retort. It would be a great gain if a cold and dispassionate survey of the facts could be made and all falseness eliminated from advocacy and prosecution.

Vast interests profit by the sale of alcoholic drinks. They naturally resent restraints, though it is but just to recognise that "The Trade" does not like men to be drunken, and is keenly alive to the fact that every man drunk is a good prohibition argument. But the interests are interested and so cannot be judicial. The men who drink alcoholic drinks and enjoy them are not likely to be candid in judgment of arguments that might lead to deprivation of the pleasure. Those to whom alcohol is the accursed thing cannot be expected to be fair. It is unfortunate that there are people who cannot take alcohol at all without consuming an amount obviously too much for them, and a greater number more that take regularly a quantity that, though less obviously too much, seriously interferes with the mental and physical efficiency of the drinkers. That it is so is but little if any stronger argument for prohibition of alcohol as an article of diet than the gluttony of some is a reason for depriving all of beef and plum pudding. It must, however, be admitted that, contrary to traditional belief, none who have made scientific enquiry attach appreciable value to alcohol as a food—that is, as something that, digested and assimilated, can be used to supply energy or to build tissue. But this again is not necessarily a cause of condemnation, or, if it be, then tea and coffee come under ban.

Most people who consume alcoholic drinks take then because they like them and enjoy a certain degree of intoxication. That is no bad reason either, but let it be given as the good reason and no excuse made. Directly a claim is made that the drink is taken for any other reason, and not because it is liked, there is a confession of doubt in the righteousness of the act. He who claims he cannot do his business unless he has a whisky or two in the course of the forenoon, and a glass or two of port after dinner, probably has never tried the effect on his work of a couple of months' total abstinence, and so little believes in the virtues of his panacea that he strongly objects to his clerks taking a nip or two of a morning. If alcohol improves work why should he object?

Any one of the working men who loudly proclaim "a man cannot get the best out of himself without beer" and clamour to the Government for a larger, stronger, and cheaper supply for "the wage earners," if he had five shillings on a football match, and learnt that the members of the team of his fancy were taking a pot of beer apiece afternoons and evenings, would curse them for a swilling crew and promptly hedge his dollar. If the manual labourer can only get the best out of himself by the help of beer, why not the professional football players? Such claims are cant and scarcely self-deception. But if a man says boldly alcohol is one of the pleasures of life and that he means to have it, who has the right to say him nay, provided that by reason of his indulgence in his pleasure neither he nor those he should support become a charge on the community?

That it is one of the pleasures of life is probably the only good excuse for the free use of alcohol; but since, though a pleasure, it is a dangerous pleasure, he who would indulge should know that and not seek to represent his indulgence as a virtue. This he does when he claims that his strength, his efficiency or his health is the better for his indulgence. It is doubtful if alcohol, except if it be taken in very large quantity, shortens life, if it does the number of years lost is perhaps small, but it gives a very long old age. It is the explanation of "too old at forty." Not too old to go on living, but too old to be much good to anybody, because thus early old age has come. A man's arteries have hardened prematurely. His joints have stiffened early. His brain does not function quickly nor readily receive new ideas. He is set in his habits. Old age and the climateric have come to the man before due time as the result of steady, daily, prolonged poisoning of alcohol. He is not too old to live, he may live another thirty years, but too old for efficiency. He may go on living but he will be an old man all the time and a long, long time quite likely.

It is the inefficiency caused by alcohol that has been the chief argument of the anti-saloon party in America. The great weighty argument has been, "So long as there are saloons you will drink, so long as you drink you will be less efficient than you might be." Simply because there has been no teetotal ranting, and very little abuse of the other party, but a steady, solid common sense propaganda, the working men of America have accepted the reasoning, have willed to be saved from temptation and have altered the constitution of the United States to give affect to their will. It is not unlikely this astonishing course of a great democracy may have more effect in shaping the destiny of mankind than the great war now so nearly ended.

